# Systemic treatment with a novel basic fibroblast growth factor mimic small-molecule compound boosts functional recovery after spinal cord injury

**DOI:** 10.1371/journal.pone.0236050

**Published:** 2020-07-17

**Authors:** Shiro Imagama, Ryoko Ogino, Shinya Ueno, Norihito Murayama, Naohiro Takemoto, Yoshiari Shimmyo, Taisuke Kadoshima, Shigeki Tamura, Mariko Kuroda, Yukihiro Matsuyama, Kenji Kadomatsu, Yasuhiro Morita, Teruyoshi Inoue, Naoki Ishiguro

**Affiliations:** 1 Department of Orthopaedic Surgery, Nagoya University Graduate School of Medicine, Nagoya, Japan; 2 Asubio Pharma Co., Ltd., Kobe, Japan; 3 Department of Orthopaedic Surgery, Hamamatsu University School of Medicine, Hamamatsu, Japan; 4 Department of Biochemistry, Nagoya University Graduate School of Medicine, Nagoya, Japan; 5 Faculty of Pharmacy, Laboratory of Physiology and Morphology, Yasuda Women’s University, Hiroshima, Japan; University of Louisville, UNITED STATES

## Abstract

Neurotrophic factors have been regarded having promising potentials for neuronal protection and regeneration, and thus promoting beneficial effects of kinesiological functions. They can be suspected to play important roles in cell/tissue grafting for various neural diseases. The clinical applications of such trophic factors to the central nervous system (CNS), however, have caused problematic side effects on account of the distinctive bioactive properties. In the course of developing synthetic compounds reflecting beneficial properties of basic fibroblast growth factor (bFGF), we conducted screening candidates that stimulate to trigger the intracellular tyrosine phosphorylation of FGF receptor and lead to the subsequent intracellular signaling in neurons. A small synthetic molecule SUN13837 was characterized by mimicking the beneficial properties of bFGF, which have been known as its specific activities when applied to CNS. What is more remarkable is that SUN13837 is eliminated the bioactivity to induce cell proliferation of non-neuronal somatic cells. On the bases of studies of pharmacology, behavior, physiology and histology, the present study reports that SUN13837 is characterized as a promising synthetic compound for treatment of devastating damages onto the rat spinal cord.

## Introduction

Spinal cord injury (SCI) is a life threatening medical condition that often leads to substantial, permanent neurological impairment. There is currently no drug treatment for SCI approved by the United States (US) or the European Union (EU) regulatory agencies, and although high-dose steroids are commonly used, there is little empirical evidence of a therapeutic benefit. The annual incidence of SCI in the US is about 13,200 to 15,600 cases per year [1], with over 50% of cases resulting in some degree of tetraplegia. The estimated lifetime costs for a 25-year-old patient with tetraplegia ranges between 1.7 and 3.1 million dollars and for patients with paraplegia around 1 million dollars [1]. Therefore, SCI is a catastrophic medical condition that imposes disproportionately large economic and social costs on its victims and society in general. Thus, investigation of the mechanisms underlying the pathology of SCI to identify therapeutic targets for SCI is among the major themes in life science, as well as pharmaceutical research. Recent research has revealed that the pathogenesis is caused by progressive and sequential events at the injury site: primary SCI with neuronal necrosis and rupture of axons is accompanied by inflammation with infiltration of neutrophils and activation of resident microglia, resulting in a secondary injury, with apoptosis of neurons and oligodendrocytes, and finally formation of a cavity and glial scar [2, 3, 4]. Considering the importance of neurotrophic factors for successful rehabilitation and the recent focus on neuronal stem cell transplantation, which is currently being tested as a treatment for SCI in preclinical studies, there is much evidence for the potential of neurotrophic signaling to improve regeneration after SCI. Accumulating research has revealed that endogenous expression of both basic fibroblast growth factor (bFGF) and FGF receptors (FGFR) are upregulated at the injury site shortly after spinal cord damage; acting to prevent cell necrosis/apoptosis and axonal growth inhibition associated with SCI pathogenesis [5]. Although bFGF exhibits potent neuroprotective activity and promotes axonal outgrowth [6, 7, 8, 9], it also has the potential to stimulate proliferation of cells responsible for either inflammation or glial scar formation at the injury site [10, 11, 12], and these opposing actions of bFGF reduce its value as a therapeutic agent for SCI. This is underscored by the fact that bFGF treatment in an animal model of SCI led only to ambiguous results and administration of bFGF to patients with brain infarction exhibited serious adverse effects, such as decreased blood pressure and increased numbers of leukocytes due to enhanced proliferation of inflammatory cells [13]. Other undesired side effects such as carcinogenesis [14] have also been reported. bFGF treatment, however, did show some advantageous outcomes in the long-term recovery phase following cerebral stroke [15]. Taken together, these data suggest that a selective activator of FGF receptors lacking cell proliferating activity would be a preferable therapeutic agent for the treatment of SCI, with the ideal candidate compound profile exhibiting all of the beneficial activities of bFGF but no proliferative effect and better pharmacokinetic properties compared with bFGF.

We performed a screen of our in house compound bank for agents that would enhance intracellular tyrosine phosphorylation of the FGFR, and then carried out hit optimization by synthesizing and evaluating a large number of compounds in multiple functional screening assays and finally identified a small molecule we named SUN13837. SUN13837 activates several signaling factors that mimic the biologically advantageous effects of bFGF, such as neurite outgrowth and neuroprotective effects, but does not induce the cell proliferation responsible for inflammation and glial scar formation at the injury site. The pharmacokinetic properties of SUN13837 are promising in terms of oral bioavailability (70%) and distributes to the brain in rats (Kp = 0.15) [16] thus implying that SUN13837 is a reasonable candidate compound for use in various diseases in which neurons are vulnerable.

In this paper we present the details of the functional bFGF mimic SUN13837, which selectively activates the FGFR without promoting cell proliferation, and argue for the therapeutic relevance of SUN13837 in SCI and other diseases of the central nervous system that currently have no approved drug treatment.

## Materials and methods

### Animal care

The study was performed at the Nagoya University Graduate School of Medicine and at Asubio Pharma, Co., Ltd. All animal procedures were approved and performed in accordance with the Nagoya University Animal Care and Use Committee (Approved No. 31086) and the Asubio Pharma Animal Care and Use Committee (Approved No. AEK-10-078 and AEK-11-207), respectively.

Female Slc: SD rats (Japan SLC, Inc.) were used at a body weight range of 180–250 g. Animals were housed in groups of 3 animals /cage with a 12:12-h light-dark cycle. Food and drinking water were supplied freely. For 10 days after surgery, tap water containing 0.2% sulfamethoxazole (Bactramin, Chugai Pharmaceutical) was supplied by water bottles to prevent infection, and manual urination was performed 1 or 2 times a day. All animals were euthanized by CO_2_ inhalation at the end of the evaluation period, except for histological studies.

### Neurite outgrowth assay

Hippocampal neurons were prepared from E18 Wistar rat embryos using the Papain Dissociation System (Worthington Biochemical Corp., NJ) and plated in Neurobasal medium containing 5% Nu-Serum and 2% B-27 at a cell density of 1×10^5^ cells/dish in 35 mm dishes coated with poly-D-lysine. Neurons were cultured at 37°C and-5% CO_2_. On DIV3 and DIV7, half of the culture medium (1 ml) was exchanged with Neurobasal medium containing 8 μM AraC, 5% Nu-Serum and 2% B-27 to inhibit the proliferation of glial cells. Then, 20 μl of 100-fold solutions of SUN13837 or bFGF (Upstate Biotechnology, NY) were added to the culture medium. HBSS containing 0.27 N HCl and 3% ethanol was used as vehicle control. On DIV9, the culture medium was removed and cells were washed twice with PBS, then fixed with phosphate buffered 2% paraformaldehyde. After another wash with PBS, cells were stained with GAP-43 antibody (rabbit anti-GAP-43, Chemicon International Inc.) and the ABC kit. 15 visual fields of dish were, and images were analyzed using Kurabo neuron outgrowth quantification software (Kurabo Neurocyte Image Analyzer V.1.0.0).

### MTT staining for evaluation of neuroprotection

Hippocampal neurons were prepared from E18 Wistar rat embryos as described above and plated in Neurobasal medium containing 5% Nu-Serum and 2% B-27 at a cell density of 5×10^4^ cells/well in 96-well plates coated with poly-D-lysine. On DIV7, PBS or 200 μM PD166866 were added to each well and 30 minutes later, 10μl aliquots of SUN13837 (2, 6 or 20 μM), bFGF (2 or 20 ng/ml) or HBSS were added. On the following day, 10 μl of a 400 μM glutamate solution or HBSS were added to each well. On DIV9, 10 μl of a-5 mg/ml MTT solution were added to each well and cells were dried overnight at room temperature. 100 μl aliquots of DMSO were added to each well to dissolve the reduced MTT and the absorbance was measured at 570 nm and 650 nm using a plate-reader (Multiskan MS, Thermo BioAnalysis Japan K.K.). The differences in the absorbance between the measured wavelengths was used as a measure of cell viability.

### Immunostaining for the detection of FGF receptor phosphorylation

L6 cells were plated in DMEM containing 10% FBS at a concentration of 0.9×10^4^ cells/well in 96-well plates and incubated for one day. Solutions of Human FGF receptor 1 (NM_015850) expression plasmid DNA (pcDNATM3.2/V5-DEST^®^ Gateway Vector, Invitrogen Corp. 0.2 μg/well) and lipofectamine 2000 (Invitrogen Corp., 0.5 μl/well) in Opti-MEM (Invitrogen Corp., 25 μl/well) were prepared separately, mixed together after incubation for 5 minutes and incubated at room temperature for another 20 minutes. Then, 50 μl of the reagent mix was added to each well. After 5h, the culture medium was changed to 100 μl/well DMEM containing 10% FBS. On DIV 3, the culture medium was changed to FBS-free DMEM and cells were incubated for 5-h before the addition of 5μl of 10 mM sodium orthovanadate (MERCK KGaA). After 40 minutes, 5 μl of SUN13837 (6.6, 22 or 66 μM) or bFGF (110 ng/ml) were added. In all the following procedures, sodium orthovanadate was present at a concentration of 1 mM. After incubation for 40 minutes, the culture medium was removed and the cells were washed with PBS, fixed with 4% PFA for 20 minutes, permeabilized with 4% PFA solution containing 0.1% TRITON X-100 for 10 minutes, washed three times with PBS for 10 minutes and blocked with PBS containing 20% BlockAce for 20 minutes. The primary antibody (anti-total phosphorylated tyrosine antibody PY20, Sigma, USA) was diluted 100 times with PBS containing 20% BlockAce and then allowed to react with the cells for 3 days at 4°C. Cells were washed three times with PBS for 10 minutes. The secondary antibody was diluted 350 times with PBS containing 20% BlockAce and allowed to react with the cells for 1 hour at room temperature, followed by washing the cells three times with PBS for 10 minutes. Fluorescent images of the cells were taken from 6 visual fields without overlapping at the central region of each well using IN Cell Analyzer (Ver. 3.3) (GE Healthcare Bio-Sciences K.K.). The areas of staining and integrated intensity (fluorescence intensity multiplied by the area of staining) in each image were digitalized using the Developer Toolbox (image analysis software for IN Cell Analyzer, GE Healthcare Bio-Sciences K.K.). The level of intracellular tyrosine phosphorylation was evaluated based on the integrated staining intensity.

### Measurement of BrdU uptake

SW1353 and Swiss 3T3 cells were purchased from ATCC (SW1352: Cat. No. HTB-94, Lot No.2056459, Swiss 3T3: Cat. No. CCL-92, Lot. No.4136021). Both cells were cultured with Leibovitz’s L-15 medium (for SW1353) or DMEM (for Swiss 3T3) containing 10% FBS. Cells were plated on 48-well plates and after one day the medium was changed to low-serum (0.1% FBS for SW1353 and 0.2% for Swiss 3T3) culture medium. SUN13837 (final concentrations of 0.3 to 30 μM), bFGF (final concentrations of 0.3 to 30 ng/ml) or HBSS as the vehicle were added to the cells and after 22 to 23 hours, BrdU labeling was performed according to the manufacturer’s instructions (Cell proliferation ELIZA, BrdU, Roche Diagnostics). After the addition of 10 μl BrdU-labeling solution and a two-hour incubation, cells were washed with PBS and then fixed with fixation solution for 30 minutes at -20°C and again, washed three times with PBS. After nuclease treatment and reaction with anti-BrdU-POD antibody, peroxidase substrate was added to each well. After color development, the absorbance at 405 nm and 490 nm was measured with a plate reader (POWERSCAN^®^ HT; Dainippon Sumitomo Pharma Co., Ltd.). The difference between the absorbance at the measurement and reference wavelengths was taken as a measure of BrdU uptake.

### Western Blot for measurement of cell cycle regulator protein levels

Swiss 3T3 cells were plated to 100-mm dishes and cultured in DMEM containing 10% FBS. After one day, the culture medium was changed to low-serum culture with DMEM containing 0.2% FBS and after two days, SUN13837 (final concentration of 10 μM), bFGF (final concentration of 10 ng/ml) or HBSS were added. After incubation for 16 h, the cells were washed with ice-cold PBS. M-PER Reagent containing 1% protease inhibitor cocktail (400 μl) was added to each dish, and cells were collected. Cells were vortexed for 10 seconds, followed by 5 min incubation on ice, vortexed again for 10 seconds and centrifuged (15,000 rpm, 15 minutes, 4°C), Supernatants were collected and protein concentrations determined using the BCA assay, and adjusted to 0.25 mg/ml. After the addition of NuPAGE LDS Sample Buffer (4X) and NuPAGE Sample Reducing Agent (10X), the supernatants were heated at 70°C for 10 minutes. The supernatant samples (10 μl per lane) were electrophoresed using three NuPAGE 4–12% Bis-Tris Gel (180 V for 65 minutes; Electrophoresis cassette, TEFCO; CrosPower, ATTO Corporation) for each sample. The electrophoresed samples were transferred to Immobilon-PSQ membranes presoaked in methanol (20 V for 120 minutes; Trans-Blot SD Semidry Transfer Cell, Bio-Rad Laboratories, Inc.). The membranes were washed 3 times with TBST and blocked with Blocking One (room temperature for 70 minutes). Each of three membranes per sample was incubated with anti-cyclin D1 (MERCK KGaA), anti-p27/kip 1 (Becton, Dickinson and Company) and anti-actin primary antibody (Santa Cruz Biotechnology, Inc.), respectively (in a low temperature room for one night). After washing 3 times with TBST, the membranes were incubated with HRP-labeled anti-mouse-IgG or anti-goat-IgG secondary antibody (at room temperature for 120 minutes). After washing 3 times with TBST, the bound HRP-labeled antibody was detected by incubation with Super Signal West Dura Extended Duration Substrate. The blotting image was then imported by Lumino Image Analyzer (LAS3000 and Multi Gauge Ver.3.0, FUJIFILM Corporation). The amount of protein expression was quantified as the density multiplied by the area of the band of the target molecular weight using Lumina Vision (Mitani Corporation).

### Surgical procedure for a rat SCI model

Experiments were carried out as previously reported [17]. In short, animals were anesthetized with an i.p. injection of a mixture of ketamine hydrochloride (Ketalar, Daiichi-Sankyo), xylazine hydrochloride (Selactar, Bayer), and ethyl carbamate (Urethane, Sigma-Aldrich). After the spinal column was exposed by dorsal midline incision, the 9th thoracic vertebral arch was removed with bone scissors, paying attention not to damage the dura mater. The exposed spinal site (T9) was kept just under the impactor device (IH IMPACTOR-I; Precision System Instrumentation) and then a 200-kdyn force (mean force: 230 kdyn, mean displacement: 1578 μm, dwell time: 0 sec) was applied to the site. Tonic extension of the hind limbs caused by the spinal cord injury was confirmed. The incision was sutured and SUN13837 (0.3 or 1 mg/kg) or vehicle was administered intravenously via the tail vein at 90 minutes or 12 hours after contusion in a volume of 1 ml/kg with pre-assigned grouping. Drug or vehicle were administered once daily for 10 days.

### Evaluation of hind limb motor dysfunction in a rat SCI model

The recovery of motor function of the hind limbs was evaluated by 2 individual investigators in a blinded manner, according to the Basso-Beattie-Bresnahan locomotor scale [18] (BBB score) once a week until 8 weeks after injury, and the mean value of both sides was employed as individual observed data. BBB scoring was performed based on spontaneous motor activity observed in a round shaped empty open field arena, 100 cm in diameter, during the light-on period. Each observation was performed once for two minutes, which is a sufficient duration to evaluate the motor function of the hind limbs.

### Motor evoked potential (MEP) measurement

After the rats were anesthetized with a mixture of ketamine and xylazine, the skull was exposed and openings were made over the sensorimotor cortex with a dental drill. Stimulation in five train pulses (60–80 mA, 0.2 msec, 1 kHz) was performed via electrodes with the anode positioned on the sensorimotor cortex and the cathode on the nasal cavity. MEPs were recorded at the contralateral tibialis anterior (TA) muscle using LabChart 7 (AD Instruments), and the average amplitude and latency of 50 traces was calculated.

### DiI-staining and tracing of neuron projections in spinal cord after SCI

At 11 weeks after spinal cord injury, a DiI (Invitrogen)-impregnated filter was inserted into the sensorimotor cortex of the hind limb control area. After one week, the animals were deeply anesthetized and perfused transcardially with 4% paraformaldehyde. The spinal cords were carefully removed, embedded in egg yolk and sections were prepared (shown in detail in S4 Fig).

### Evaluation of axon regeneration beyond the contusion site

Animals for which behavioral evaluation (Fig 4C) has ended were deeply anesthetized and perfused transcardially with 4% paraformaldehyde. The spinal cords were carefully removed, paraffin-embedded sections were prepared. The paraffin sections were incubated with anti-5-HT primary antibody (Imunostar, Inc. 20080), subsequently treated with VECTASTAIN Elite ABC Rabbit IgG Kit, and then developed with diaminobenzidine.

5-HT-positive area at an epicenter and at 4 mm each at the rostral and caudal sides were measured by ImageJ [19, 20] (Ver.1.43) and expressed as an area ratio.

The method of image processing is as follows.

- The spinal cord sections prepared by the above method was captured into ImageJ.
- The captured image is divided into RGB colors.
- A 5-HT positive image was created by subtracting from the red image with the blue image as the background.
- The 5-HT-positive area of the obtained image was measured and expressed as a percentage of the tissue area.

### Statistical procedures

All data are reported as the mean with standard error. No statistical methods were used to predetermine the sample sizes in the *in vitro* studies. In the animal studies, rats were randomized into 3 groups, in order to minimize the average and the standard deviation of their body weight by SAS Ver.9.1.3 (SAS Institute Inc.).

The parametric two-tailed Dunnett type multiple comparison, two-tailed Student’s t-tests or two-tailed Welch’s t-test were performed relative to the control group. When the p-value was less than 0.05 (p < 0.05), the difference was judged to be significant. Statistical analyses were performed with SAS Ver.9.1.3 (SAS Institute Inc.).

## Results

### Neurite outgrowth induction by SUN13837 and bFGF

**Fig 1:**
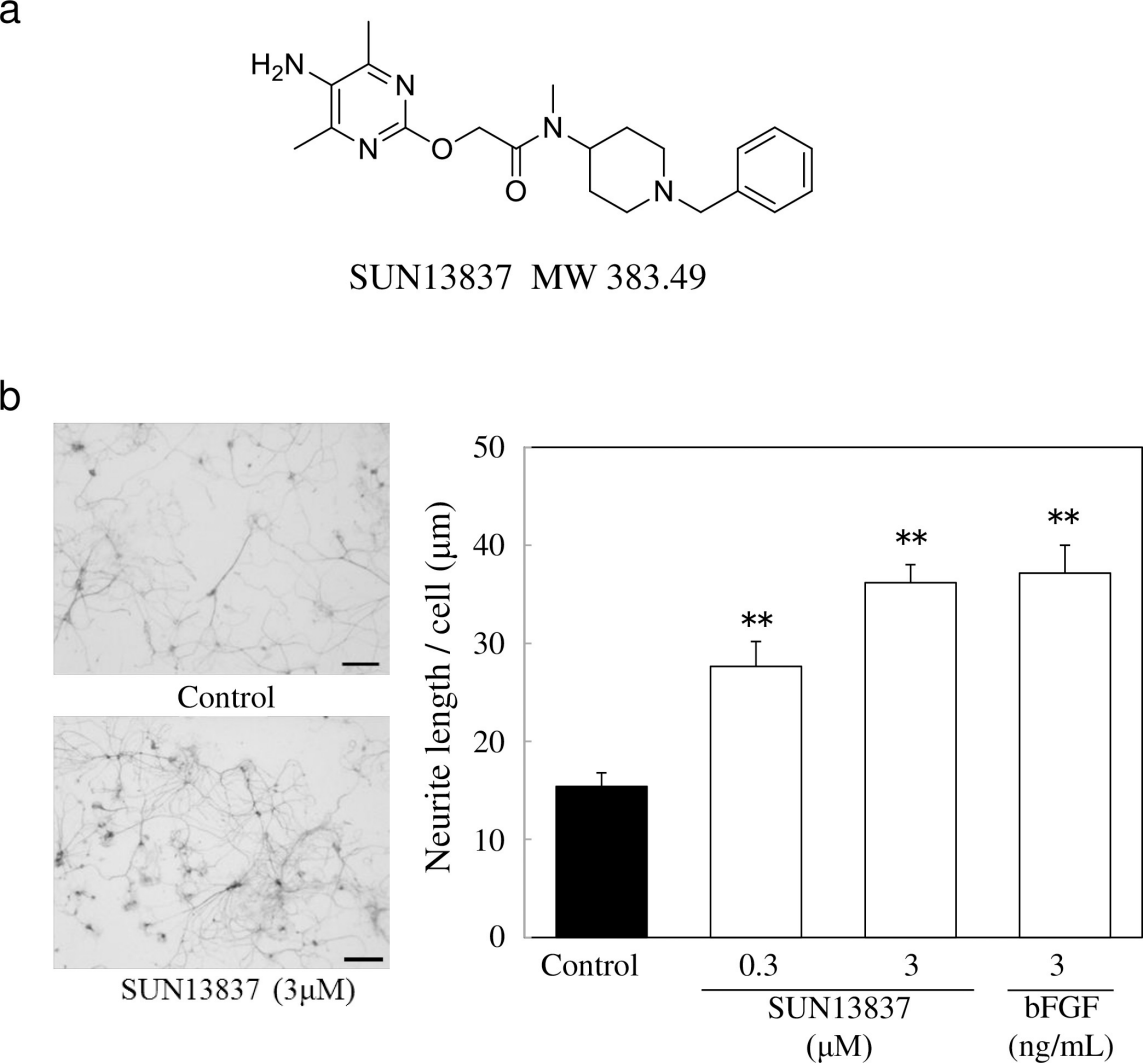
Effect of SUN13837 and bFGF on neurite outgrowth in primary cultures of rat hippocampal neurons. a, Chemical structure of SUN13837. b, Representative photomicrographs of neurite outgrowth in cultured rat hippocampal neurons treated with vehicle (top) or 3 μM SUN13837 (bottom). Scale bar represents 100 μm. Quantification of neurite outgrowth effect of SUN13837- or bFGF-treatment. Data are expressed as the mean ± SEM (n = 15) ** p < 0.001, vs. control by two-tailed Dunnett’s test.

The effects of SUN13837 (2-([5-Amino-4,6-dimethylpyrimidin-2-yl] oxy)-N-(1-benzylpiperidin-4-yl)-N-methylacetamide, Fig 1A), and bFGF on neurite outgrowth were examined in primary cultures of rat hippocampal neurons. Because neurite outgrowth might only be a secondary effect following improved neuronal survival, we examined neurite length per cell body, using the IN Cell Analyzer 1000 with the neurite outgrowth detection program. As shown in Fig 1B, SUN13837 treatment induced neurite outgrowth in primary neurons. The effect of SUN13837 was significant at all concentrations tested and 3μM SUN13837 showed an effect equivalent to that of 3 ng/ml bFGF (Fig 1B).

### Neuroprotection and FGFR-1 phosphorylation by SUN13837 and bFGF

**Fig 2:**
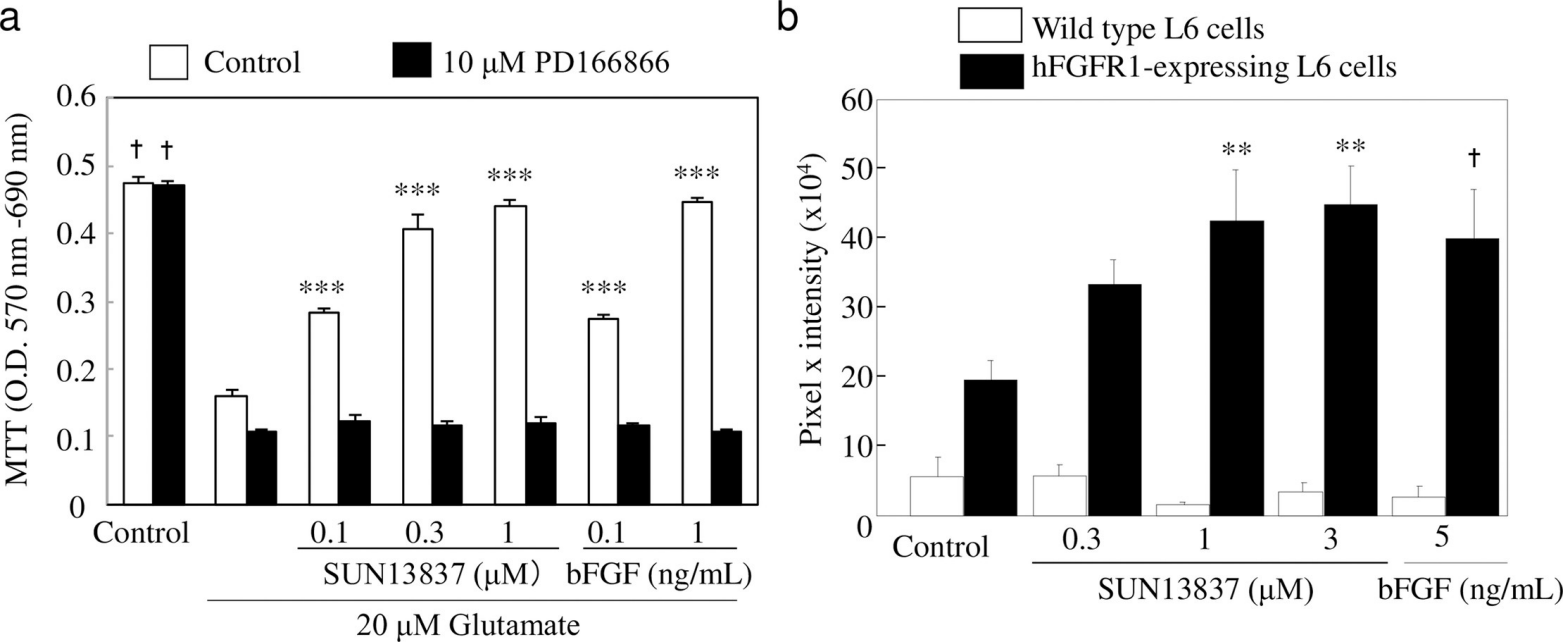
Effect of SUN13837 and bFGF on glutamate-induced cell death and intracellular FGFR-1 tyrosine residue phosphorylation. a, Protective effects of SUN13837 and bFGF against glutamate-induced neuronal cell death measured by MTT staining in primary cultures of rat hippocampal neurons in the presence or absence of 10 μM PD166866. b, Intracellular FGFR-1 tyrosine residue phosphorylation levels after SUN13837 or bFGF treatment in wild-type or human FGFR-1 expressing L6 cells. Quantification of immunostaining with a total phospho-tyrosine antibody is shown. Data are expressed as the mean ± SEM (n = 6 for a, n = 12 for b), *** P<0.001, ** p<0.01 vs. glutamate (a) or control (b) by two-tailed Dunnett’s test. ✝ p<0.05 vs. glutamate (a) or control (b) by two-tailed Student’s t-test.

Stimulation of primary cultures of hippocampal neurons with SUN13837 or bFGF prevented glutamate-induced neuronal death (Fig 2A). Since SUN13837 mimics the neuroprotective activities of bFGF, it was important to determine whether SUN13837 could activate intracellular signaling mediated by FGFR1, which is one of the main bFGF receptors in neurons. For this purpose, we examined the neuroprotective effects of SUN13837 in the presence of PD166866 [21], a specific inhibitor of the FGFR-1 tyrosine kinase. The neuroprotective effects of SUN13837 and bFGF were completely antagonized when 0.3 μM PD166866 was added to cultures of hippocampal neurons 30 min before the application of SUN13837 and bFGF (Fig 2A).

For clinical application, it is crucial to confirm that the human FGFR is phosphorylated by SUN13837. In wild-type L6 cells, a cell line derived from rat skeletal muscle that lacks FGFR expression, neither bFGF nor SUN13837 showed any effect on intracellular tyrosine phosphorylation. However, in L6 cells transfected with human FGFR-1, both bFGF and SUN13837 significantly enhanced intracellular tyrosine phosphorylation (Fig 2B).

### Differential activities of SUN13837 and bFGF on cell proliferation

**Fig 3:**
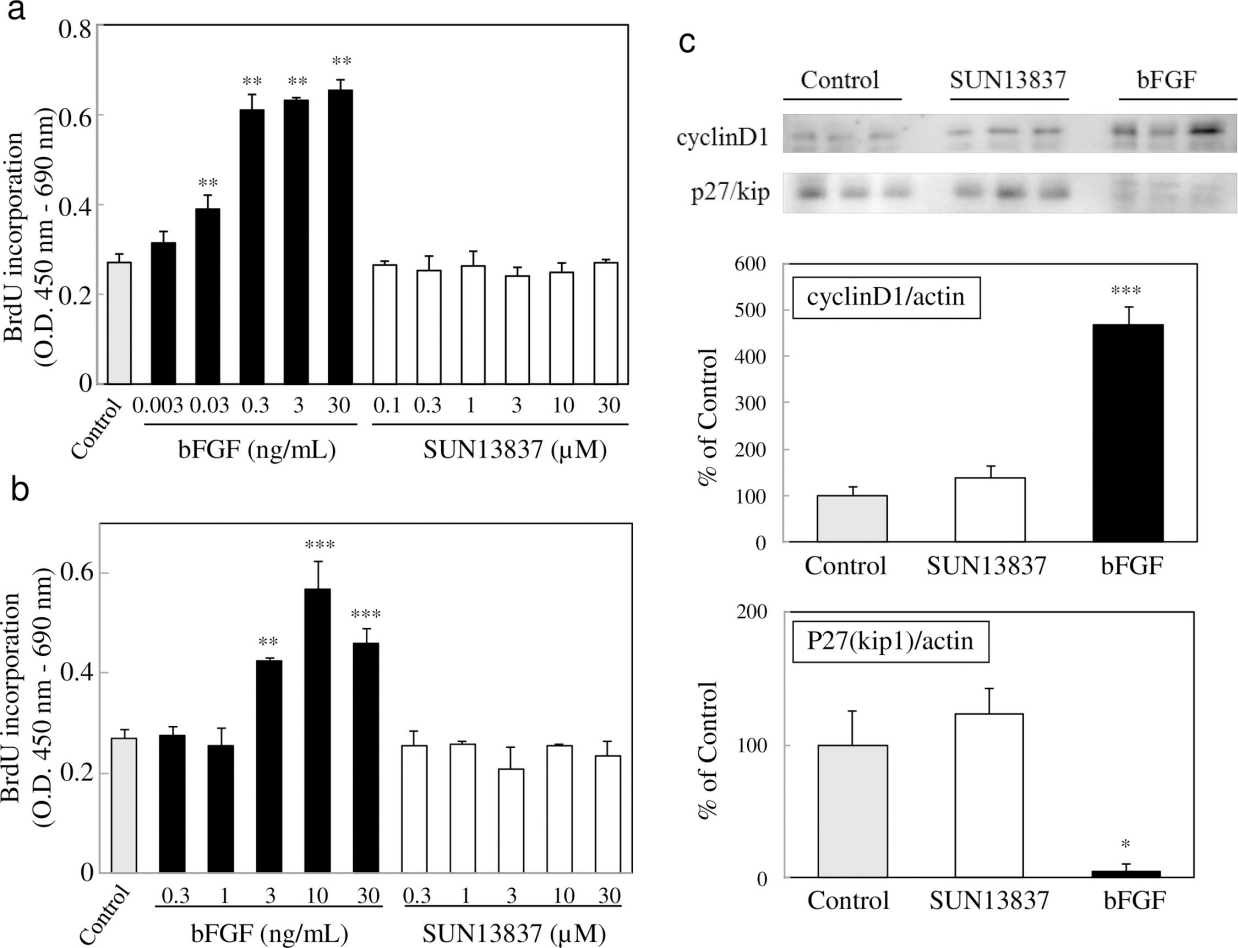
Effects of SUN13837 and bFGF on cell proliferation. a, b, Cell proliferation with SUN13837 and bFGF measured by BrdU incorporation in SW1353 (a) and Swiss 3T3 (b) cells. Data are expressed as the mean ± SEM (n = 4). *** p<0.001, ** p<0.01 vs. control by two-tailed Dunnett’s test. c, Immunoblots of cyclin D1 and p27 (kip1) protein expression in Swiss 3T3 cells after treatment with 10 μM SUN13837 or 10 ng/ml bFGF. Quantification of immunoblots is shown below. The expression of cyclin D1 and p27 (kip1) was normalized to actin expression levels. Results are shown as percentage of protein expression relative to control. Data are expressed as the mean ± SEM (n = 3). *** p<0.001, * p<0.05 vs. control by two-tailed Dunnett’s test.

Another important issue is whether SUN13837 exhibits the same proliferative activity as bFGF. We investigated this issue using the human chondrosarcoma cell line SW1353 and the Swiss mouse embryo fibroblast cell line Swiss 3T3, which have been confirmed to proliferate in response to bFGF stimulation [22], by measuring BrdU incorporation into mitotic cell nuclei. While bFGF markedly increased BrdU incorporation into these cells (Fig 3A and 3B), stimulation with SUN13837 up to 30 μM yielded no significant increase in BrdU-labeled SW1353 and Swiss 3T3 cells (Fig 2A). This demonstrates a clear difference in cell-proliferative activity between SUN13837 and bFGF.

bFGF-induced cell proliferation requires the activation of an intranuclear cell proliferation signal. To examine whether SUN13837 exhibits the same effects as bFGF on intranuclear protein expression, expression levels of the cell cycle regulators cyclin D1 and p27 (kip1) as markers of cell proliferation were compared in Swiss 3T3 cells. bFGF increased cyclin D1 expression by 4.6-fold and decreased p27 (kip1) expression to 5% of control levels (p = 0.0203 and p = 0.0002, respectively, Fig 3C and 3D), consistent with the previous findings [23]. In contrast, SUN13837 did not exhibit any effect on the expression levels of these cell proliferation markers (p = 0.6089 for cyclin D1 and p = 0.5803 for p27 (kip1), Fig 3C and 3D), indicating that there are substantial differences in the action on cell proliferation between SUN13837 and bFGF.

### Effect of SUN13837 on hind limb motor dysfunction in the rat SCI model

**Fig 4:**
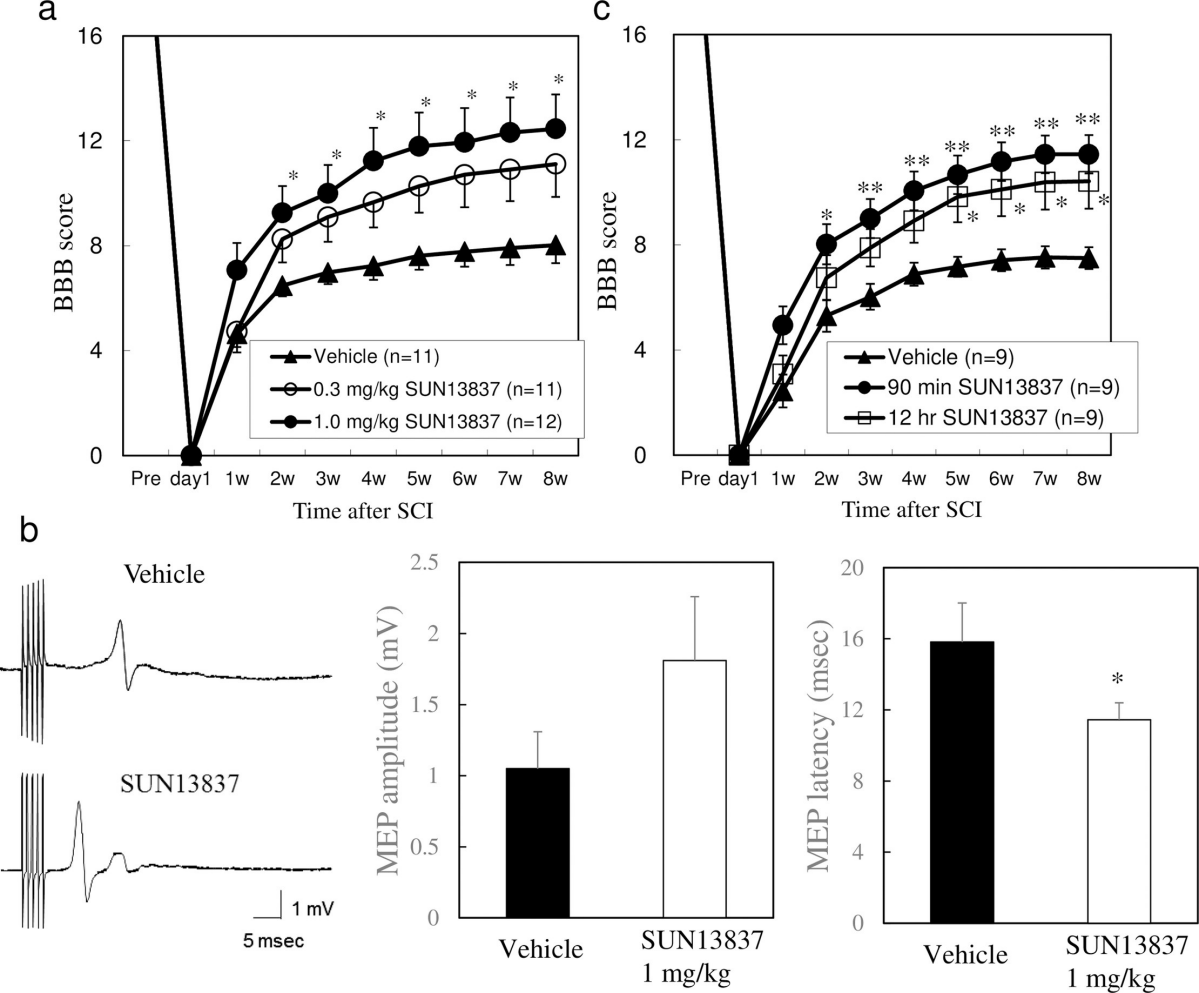
Effect of SUN13837 on locomotor function in rat SCI model. a, Effect of SUN13837 on BBB score with the first SUN13837 administration 90 min after injury. Data are expressed as the mean ± SEM (n = 11 to 12). b, Motor evoked potential (MEP) measurements 12 to 13 weeks after injury in rats treated with vehicle or 1 mg/kg SUN13837 90 min after injury. Data are expressed as the mean ± SEM (n = 17 for vehicle and 19 for SUN13837). c, Effect of 1 mg/kg SUN13837 on BBB score in rat SCI model the first administration 90 min or 12 h after injury. Data are expressed as the mean ± SEM (n = 9). * p<0.05, ** p<0.01 vs. vehicle by two-tailed Dunnett’s test for behavior assessment and two-tailed Student’s t-test for MEP.

In all of the animals used in the SCI model, complete paralysis of the hindlimbs (BBB score = 0) was confirmed one day after injury. 8 weeks after injury, the score of the vehicle-treated group had improved to a score of 8 (Fig 4A). In the drug-treatment groups, SUN13837 was administered intravenously once daily for 10 days starting from 90 minutes after injury. In the 1 mg/kg SUN13837 treatment group, significantly enhanced recovery of hindlimb motor function was observed from 2 weeks after surgery onwards, with the BBB score exceeding 12 at 8 weeks after injury (p = 0.017), whereas treatment with 0.3 mg/kg SUN13837 increased the BBB score compared with the vehicle-treated group at all time points, but did not reach significance (p = 0.118, Fig 4A). There were no adverse events observed after intravenous administration of SUN13837.

After the end of behavioral observation, electrophysiological evaluation was performed. The latency of the motor evoked potential (MEP) onset in the hind limb muscles, which reflects the function of spinal cord descending tracts, was increased in animals with SCI, but treatment with 1 mg/kg SUN13837 significantly shortened the MEP latency compared with vehicle-treated animals (p = 0.0146, Fig 4B). This indicates that neuronal connectivity to the hind limbs is improved by SUN13837 treatment.

We next investigated the therapeutic time window during which post-injury treatment with SUN13837 was effective. In a group which received the initial dose of 1 mg/kg SUN13837 at 12 hours after injury, the recovery of motor function was significantly enhanced from 5 weeks onwards compared with the vehicle-treated group (p = 0.003 for 90min, p = 0.025 for 12hr at 8 week, Fig 4C). These results indicate that the therapeutic time window of SUN13837 in the treatment of SCI is at least 12 hours.

### Promotion of axon regeneration of the corticospinal tract (CST) by SUN13837

**Fig 5:**
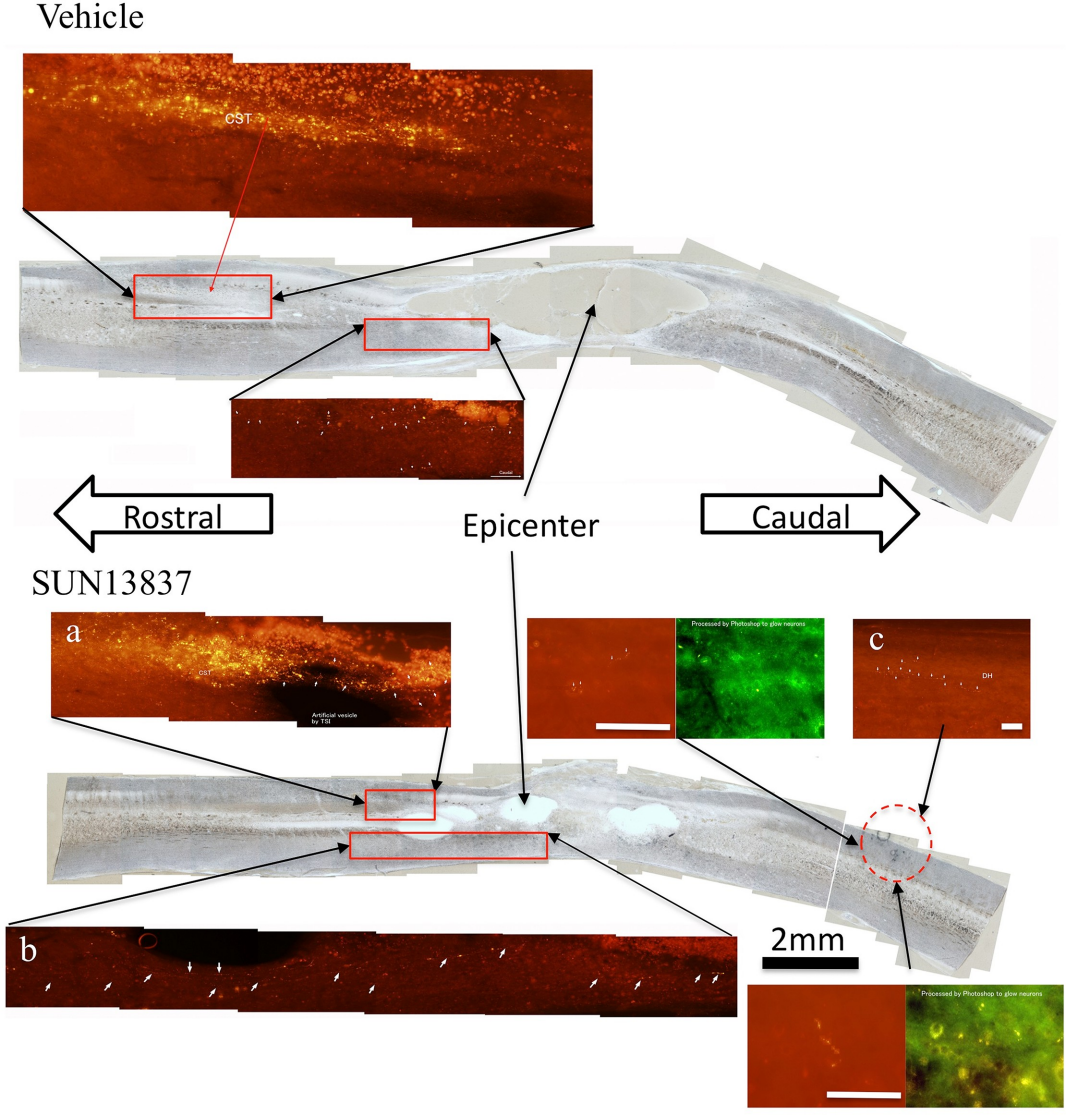
Representative pictures of anterograde axonal tracing in vehicle- or SUN13837-treated rats. Top, Vehicle-treated: A few DiI-labeled axons traveled for a very limited distance in the ventral funiculus of the spinal cord, which was slightly affected by the contusion lesion. This implies that the corticospinal tract neurons appeared to possess innate powers of regeneration even in control animals with SCI. Bottom, SUN13837-treated: DiI-labeled axons traveled caudally for a long distance in the ventral funiculus of the spinal cord; where in normal rats the corticospinal tract axon did not take the course for the spinal projection. A number of DiI-labeled axons were traced to the rostral border of the intact lumbosacral spinal cord in SUN13837-treated animals. Another group of DiI-labeled axons were traced caudally through the wounded tissues of the dorsal spinal cord into the uninjured dorsal horn of the lumbosacral spinal cord. The axons sprouted from distal stumps of the injured corticospinal tract appeared to complete their regeneration by exhibiting axonal arborizations around intrinsic neurons in the dorsal horn of the lumbosacral spinal cord. White scale bar represents 200 μm. Since the Red image contains not only DiI fluorescence but also intrinsic fluorescence, the intrinsic fluorescence is shown as Green image. In order to facilitate identification of the DiI-labeled axons, magnified photos including a, b, and c in the figure were presented in the supplemental section (S6 Fig).

In hodological study, the effect of SUN13837 on regeneration of CST axons was investigated by anterograde axonal tracing of the lipophilic fluorescent dye DiI (1,1'-dioctadecyl-3,3,3',3'-tetramethylindocarbocyanine perchlorate) after application to the hind limb region of the cerebral motor cortex (S4 Fig) in rats used in the behavioral studies. DiI-labeled axons were observed traveling caudally in the most ventral portion of the contralateral dorsal funiculus of the spinal cord (i.e., the native course of rat CST). Intense labeling was traced caudally to the distal stumps of severed CST axons (i.e., the distal axonal ends of the survived CST neurons in the contralateral cerebral cortex) in either the vehicle- or SUN13837-treated rats. In SUN13837-treated rats, furthermore, many DiI-labeled axons arising in the distal stump of the CST continued to proceed caudally through the contused tissues, either neural or unidentifiable damaged tissues, and then to enter the lumber and sacral spinal segments caudal to SCI. Those labeled-axons were observed primarily in the ipsilateral dorsal halves of the contused spinal cord through their entire courses. Some groups of DiI-labeled axons were able to be traced successfully into the normal dorsal horn of the lumbo-sacral spinal cord (Fig 5). The DiI-labeled axons were observed entering the dorsal horn directly and weaving around dorsal horn neurons like axon terminals. No DiI-labeled axons re-entered the CST of the caudally located spinal cord. Another group of DiI-labeled axons from the CSTs traveled ventrally and coursed caudally in the injured ventral funiculus (i.e., aberrant trajectories not seen in normal rats) but were not traced beyond the contused tissues. On the other hand, DiI-labeled regenerating axons were for the most part not observed in vehicle-treated rats in vehicle-treated rats DiI-fluorescence was exclusively restricted at sites of distal stumps of amputated CST axons, indicating halts of caudal extension of regenerating axons from survived cortical CST neurons (Fig 5, S6 Fig).

**Fig 6:**
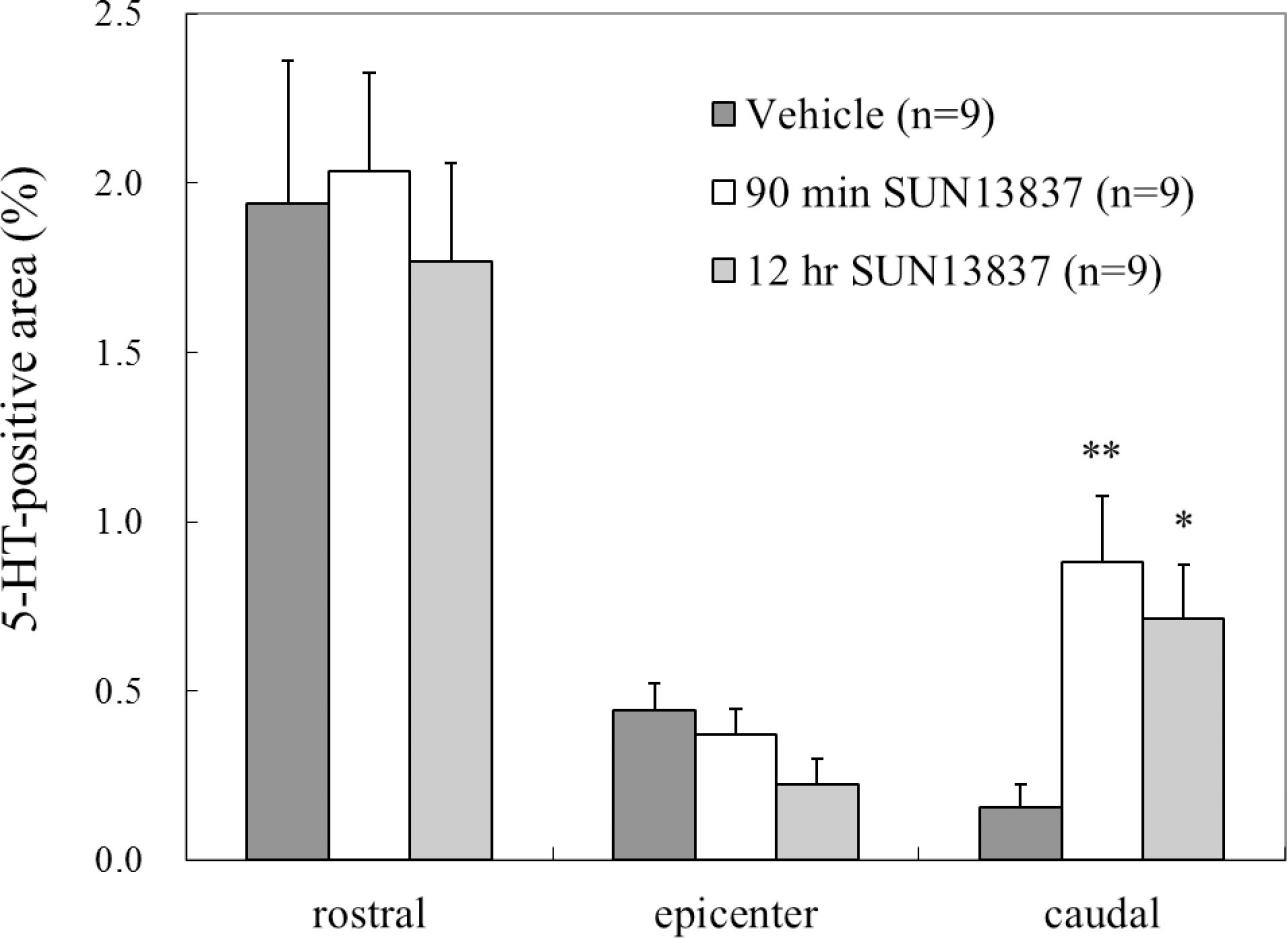
Effect of SUN13837 on regeneration of 5-HT axons. Regeneration of axons beyond the spinal cord around contusion site was detected and evaluated using an antibody for 5-HT (5-hydroxytryptamine) transport ability to the periphery of the contusion site. The ratio of the number of 5-HT-positive area at 4 mm caudal side was evaluated. In the animals treated with SUN 13837 (Fig 4C), the 5-HT-positive area on the caudal side was significantly increased compared with the vehicle-treated animals, indicating that axons were elongated or sprouted by SUN13837 treatment (means ± SEM, n = 9). *p = 0.027, ** p = 0.0043 vs. vehicle-treated animals by two-tailed Dunnett’s test.

In addition, 5-HT (5-hydroxytryptamine) immunoreactivity was employed for another marker to know the tissue conditions following SCI. Immunoreactive products of 5-HT were observed in the affected regions as well as the caudally located spinal cord. This means that the tissue conditions in the contusion sites and the more caudal spinal cord has been restored to some extent as indicated by establishment of 5-HT neuronal network. In comparison with vehicle-treated rats, SUN13837-treated rats displayed significantly more amount of 5-HT immunoreactivities in the caudal lumbo-sacral spinal cord (p = 0.0043 for 90min, p = 0.027 for 12hr, Fig 6). This may be supportive for SUN13837 to re-grow in either the contused or the normal spinal cord tissues.

## Discussion

### Characteristics of a novel bFGF mimic small-molecule compound (SUN13837) superior to bFGF

The neuroprotective effects of SUN13837 and bFGF were completely antagonized when PD166866 was added to cultures of hippocampal neurons 30 min before application of SUN13837 and bFGF (Fig 2A). While a possible interaction of PD166866 with the intracellular signaling of other growth factors was also examined, the neuroprotective effects mediated by other growth factors were not affected by the presence of PD166866 (S3 Fig). Because the phosphorylation of FGFR-1 by SUN13837 or bFGF, either directly or indirectly, is linked to the neuroprotection of endangered neurons *in vitro*, phosphorylation of the cytosolic tyrosine kinase domain of the receptor must be indispensable for the initiation of the neuroprotective processes involved in the intracellular signaling of SUN13837 and bFGF (S3 Fig).

In the FGFR-1-transfected L6 cells, tyrosine phosphorylation levels in the unstimulated control group were approximately 3.5-fold higher compared with the wild-type L6 cells (control group) (Fig 2B). One possible explanation is that excessive overexpression of receptors might lead to partial receptor dimerization in a ligand-independent manner, resulting in tyrosine phosphorylation of the receptor itself as well as downstream proteins. The enhancing effect of SUN13837 and bFGF on intracellular protein tyrosine phosphorylation in L6 cells transfected with human FGFR-1 suggests that SUN13837 can activate intracellular signaling through the human FGFR-1 and implies that SUN13837 might also be effective in humans.

It was indicated that these effects were enhanced by intracellular phosphorylation similar to bFGF *via* stimulation of FGF receptors, but unlike bFGF, it does not affect the levels of intracellular cell cycle regulators cyclin D1 and p27 (kip1). According to previous studies, bFGF-induced cell proliferation requires that bFGF/FGFR1 complexes interact with Translokin/CEP57 in the cytoplasm for translocation into the nucleus, because bFGF itself lacks nuclear transport signals, and these translocated complexes then trigger cell proliferation [24, 25, 26]. This mechanism has been verified using mutated bFGF that cannot couple with Translokin or translocate into the nucleus; thus, mutated bFGF is unable to initiate mitogenic activity. However, mutated bFGF can induce cell differentiation by binding and phosphorylating FGFR1 and subsequently activating ERK1/2 [27]. This shows that the bFGF-like cell proliferation signal requires the activation of an intranuclear cell proliferation signal. To examine whether SUN13837 exhibits the same effects as bFGF on intranuclear protein expression, expression levels of the cell cycle regulators cyclin D1 and p27 (kip1), as markers of cell proliferation, were compared in Swiss 3T3 cells. Mammalian cell cycle is strictly regulated by the synthesis and degradation of cyclins and by cyclin-dependent kinases (CDK), which associate with and are activated by cyclins [28]. The D-type cyclins, including cyclin D1, are the first cyclins to be induced as G0/G1 cells are stimulated to proliferate. They are called growth factor sensors because these expressions largely depend on growth factors [29]. p27 (kip1) is one of the endogenous CDK inhibitors, which regulates the cell cycle at the G1-S transition. p27 (kip1), and remains at a high level during the G0/G1 phase, but not at the late G1 phase, the protein degrades through the ubiquitin-dependent pathway, etc., in response to mitogenic stimulation [30, 31]. It has been reported that bFGF increases cyclin D1 expression and decreases p27 (kip1) expression [23], which were also confirmed by the present results in Swiss 3T3 cells. In contrast, SUN13837 had no effect on the expression of these proteins, indicating that there are substantial differences in the action on cell proliferation between SUN13837 and bFGF.

Although we have not examined the phosphorylation rate of FGFR, we consider that the phosphorylation of FGFR by SUN13837 proceeds at the equivalent rate as bFGF. Phosphorylation by SUN13837 is as rapid as bFGF when the downstream signal of FGFR, pERK 1/2 (pERK) is observed over time. Therefore, it is considered that phosphorylation of FGFR and its subsequent activation proceed rapidly in SUN13837. However, the induction of pERK by SUN13837 does not last as long as bFGF. A similar tendency was observed in the study of Akt phosphorylation conducted at the same time, suggesting that SUN13837 and bFGF share FGFR and downstream signaling, but different activation durations. This is one of the key differences in the biological activity of bFGF and SUN13837. This point seems to suggest that some biological outcomes of both substances are different.

### Therapeutic effect of intravenous administration of SUN13837 after SCI

From our *in vitro* data, we concluded that SUN13837 promotes cellular differentiation including axonal outgrowth and confers neuronal protection *via* phosphorylation of the cytosolic tyrosine kinase domain of FGFR-1, but unlike bFGF exhibits no proliferation-inducing effect. Since it has been reported that bFGF promotes functional recovery after neuronal damage to some extent [32, 33], a higher therapeutic effect might be expected for SUN13837. Therefore, we investigated the effect of intravenous administration of SUN13837 on neuronal dysfunction in a model of rat SCI by evaluation of locomotion recovery using the Basso, Beattie, and Bresnahan locomotor rating scale [18] (BBB scale) and electrophysiological analysis.

It has been reported that bFGF attenuates the expansion of secondary damage and promotes functional recovery after neural injury by its multiple effects such as neuroprotection, differentiation of neural stem cells, neurite outgrowth and angiogenesis [32, 34]. However, due to its cell-proliferative activity, bFGF has been also reported to enhance the inflammatory reaction in the acute phase and to increase the glial scar formation around the site of injury in the chronic phase, resulting in the aggravation of pathological conditions in SCI [10, 11, 12]. On the other hand, it is suggested that bFGF may suppress the expression of GFAP *in vitro*, suppress the activation of astrocytes, and reduce pto-inflammatory cytokine level to contribute to excess astorogliosis and glial scarring after nerve injury [35]. It has also been reported that bFGF treatment suppresses the expression of TNF-a at the lesion site, decreased gliosis, and remarkably decreased the levels of chondroitin sulfate proteoglycans (CSPGs) in glia in the in vivo mouse spinal cord injury model [36].

In our preliminary study suggested that there are no difference with the tissue sparing (S7 Fig) and number of GFAP-positive cells around the epicenter (epicenter, 4 mm rostral and caudal site) between the SUN13837- and the vehicle-treated group. Since SUN13837 is a low-molecular compound possessing bFGF-like actions without causing cell proliferation. SUN13837 could exert only desirable pharmacological activities from the various actions of bFGF. In the rat SCI model used in the present study, a strong impact force of 200 kdyn was applied to the spinal cord, and to our knowledge there had been no reports of compounds showing motor functional recovery and neuronal regeneration without adverse events by intravenous injection in such a severe SCI model. SUN13837 administered intravenously once daily for 10 days starting from 90 minutes after injury showed markedly enhanced recovery of motor function of hind limbs assessed by the BBB score in the present study. MEP data also supported functional recovery. In clinical use, drug administration in a short time after SCI sometimes difficult. Then we designed the present experiment and confirmed that the 12-hour delayed treatment still showed significant recovery of motor function of hind limbs, while the recovery was less than the 90 minute-delayed treatment. Thus, these results indicated that SUN13837 has a therapeutic time window of at least 12 hours.

However, this dramatic effect were not observed in humans. SUN13837 already ended the Ph2 clinical trial [37] and unfortunately did not achieve the primary endpoint. Although we did not have the clinical results expected from the results of this paper, there are several possible reasons. In clinical trials of our compounds, we set entry criteria up to 12 hours from the onset of animal experiments, but it was difficult to meet this criteria and achieve sufficient patient entry. In a situation where a sufficient number of cases cannot be obtained, it is not easy to homogenizing the severity of spinal cord injury, so it is difficult to obtain consistent results as achieved by animal experiments.

Contusive SCI is the most common type of spinal cord injury. The present study employed a 200 kdyn contusion rat model to reproduce SCI with different severities in clinical cases. In this model, the lumbar spinal cord received devastating damages *in situ*, in particular in dorsal and central regions, with minor differences in severity of damage. Microscopic analyses constantly evinced cavitation of tissues, massive glial scars and traces of inflammation reactions in regions of SCI: that is, this rat model can be acceptable as an ideal one. SUN13837-treated rats exhibited far long-range extensions of DiI-labeled axons from the distal stumps of damaged CST, which traveled through the contused regions into the caudally located, normal lumbo-sacral spinal segments, and prevailed against the vehicle-treated rats on this point of neurite outgrowth (i.e., axonal regeneration).

The descending serotonergic pathway, which originates in brainstem neurons, plays an important role in initiating and modulating locomotion in the spinal cord [38, 39, 40]. Therefore, we attempted to quantify nerve regeneration after spinal cord injury by staining 5-HT neurons. As shown in the Fig 6, in the SUN13837 treatment group, a significant increase in 5-HT positive area was observed 4 mm distal to the contusion site (epicenter) compared to the vehicle group. This result suggests that SUN13837 contributes to the recovery from spinal cord injury by promoting the neuro-regeneration with the extension of nerve axons observed by the tracing of DiI.

In summary, SUN13837 revealed the promising properties of the neuroprotection and neurite outgrowth in our rat model of SCI, and what more the noteworthy advantage of this compound is endowed with nature lacking proliferative activity on peripheral non-neural tissues. Furthermore, our rat model demonstrated the pharmacological efficacy of SUN13837, with a wide range of therapeutic time window (TTW) and effectiveness of systemic administration. These things appear to verify successfully the data of behavioral scores and motor functions, which were provided in the previous sections. In addition, SUN13837 has been confirmed to show good bioavailability (>50%) and rapid brain distribution in rats (distribution ration is ca. 20%).

The present study has reported that SUN13837 exhibits several promising properties for neuroprotection (survival activity) and neurite outgrowth (regeneration activity) in SCI, and SUN13837 appears to be crucial in in vivo use, in particular in emergency cases, to help damaged CNS neurons survive as well as to protect CNS neurons against detrimental environments derived from neurological ailments.

## Supporting information

S1 Fig Synthesis of SUN13837. (PDF)

S2 Fig Effects of PD166866 on the neuroprotective activity of several growth factors against glutamate-induced toxicity in primary neurons. (PDF)

S3 Fig Effect of SUN13837 and bFGF on autophosphorylation sites of FGFR1 in rat primary neurons. (PDF)

S4 Fig Schematic drawing of the anterograde axonal tracing method. (PDF)

S5 Fig Immunoblotting membrane images of cyclin D1 and p27 (kip1) protein expression. (PDF)

S6 Fig Magnified axonal tracing image with DiI in rat spinal cord injury model. (PDF)

S7 Fig Effects of SUN13837 treatment on spinal cord tissue sparing. (PDF)
