# Supplementary material for: Systemic treatment with a novel basic fibroblast growth factor mimic small-molecule compound boosts functional recovery after spinal cord injury
Source: PLoS One. 2020 Jul 17;15(7):e0236050. doi: 10.1371/journal.pone.0236050 (PMC7367485; doi:10.1371/journal.pone.0236050)
Supplement: S1 Fig — (PDF) [file pone.0236050.s001.pdf]

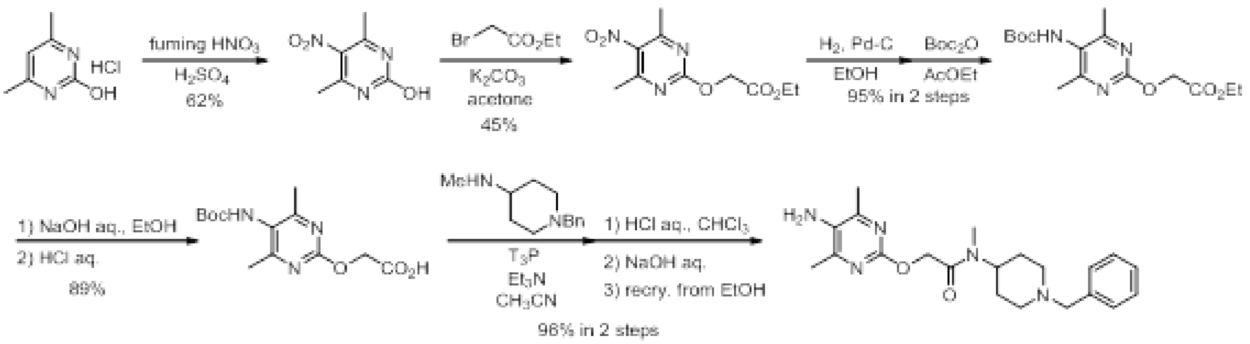

Scheme 1

S1 Fig. Synthesis of SUN13837. Reagents and conditions: The synthesis of SUN13837 was accomplished by utilizing methods similar to the patent (WO2008139894A1: Scheme 1). Characterization. 1H nuclear magnetic resonance (NMR) spectra was recorded using an ARX400 400 MHz spectrometer (Bruker BioSpin K.K., Osaka, Japan). Chemical shifts are reported in δ (ppm) units using 1H (residual) signals with tetramethylsilane used as an internal standard. Data are reported as follows: chemical shift, multiplicity (s = singlet, m = multiplet, brs = broad singlet) integration. Mass spectra were obtained using a JEOL MS Station JMS-700 in fast atom bombardment (FAB) mode.

SUN13837 2-[(5-Amino-4,6-dimethylpyrimidin-2-yl)oxy]-N-(1-benzylpiperidin-4-yl)-N-methylacetamide 1H NMR (CDCl3, 400 MHz) δ 1.54-2.14 (6H, m), 2.31 (6H, s), 2.83-3.04 (2H, m), 2.85 & 2.93 (3H, s), 3.21 (2H, brs), 3.49 & 3.51 (2H, s), 3.57 & 4.45 (1H, m), 4.90 & 4.96 (2H, s), 7.22-7.38 (5H, m). MS: m/z 384 [MH]+. mp: 164°C-166°C.
