# Supplementary material for: Systemic treatment with a novel basic fibroblast growth factor mimic small-molecule compound boosts functional recovery after spinal cord injury
Source: PLoS One. 2020 Jul 17;15(7):e0236050. doi: 10.1371/journal.pone.0236050 (PMC7367485; doi:10.1371/journal.pone.0236050)
Supplement: S2 Fig — (PDF) [file pone.0236050.s002.pdf]

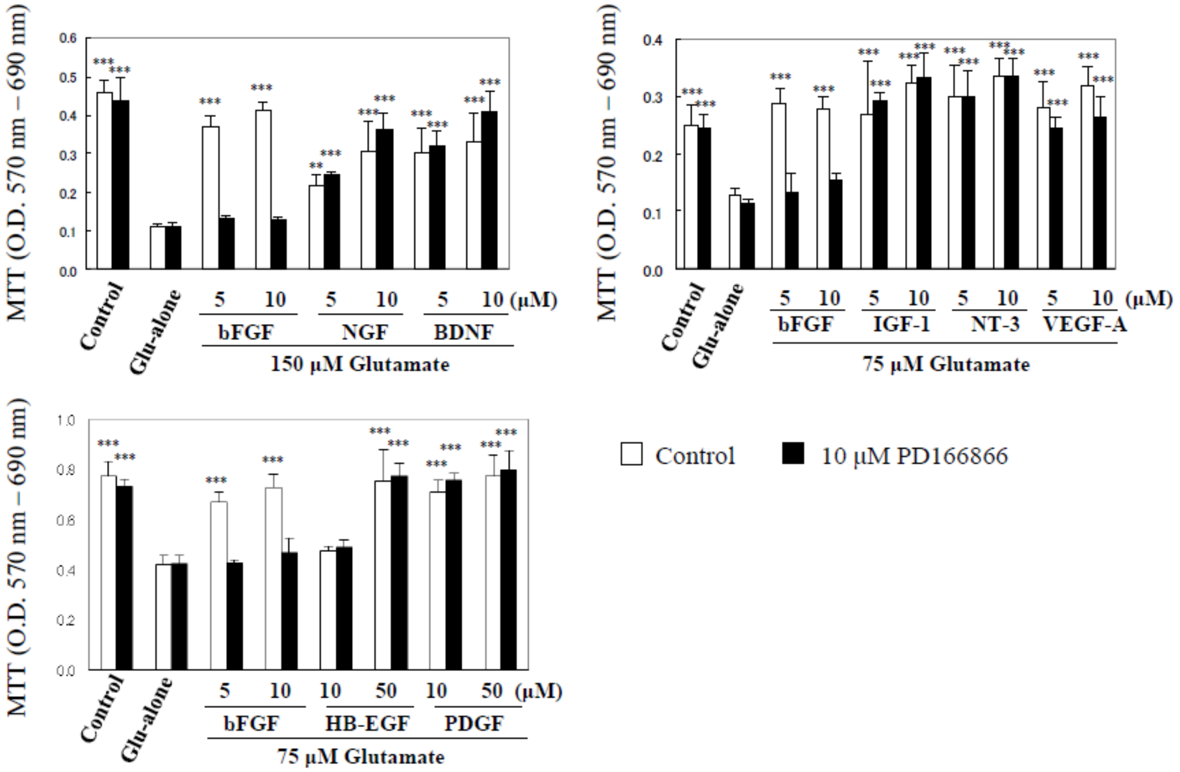

S2 Fig. Effects of PD166866 on the neuroprotective activity of several growth factors against glutamate-induced toxicity in primary neurons. Neuroprotective effects of bFGF were abolished by pretreating the cultures with 0.3 μM PD166866. This indicates that bFGF activate their neuroprotective mechanisms through the phosphorylation of the FGF receptor-1 (FGFR-1). Other growth factors (nerve growth factor [NGF], brain-derived neurotrophic factor [BDNF], insulin-like growth factor-1 [IGF-1], neurotrophin-3 [NT-3], vascular endothelial growth factor-A [VEGF-A], heparin binding-EGF [HB-EGF], and platelet-derived growth factor [PDGF]) were investigated in order to discriminate the important factors in the intracellular signaling of bFGF. Unlike bFGF, the bioactive effects of the other growth factors were not affected by PD166866. The neuroprotective mechanisms of SUN13837 and bFGF appear to differ from those of the other growth factors. The toxic concentrations of glutamate in the figures

(150 or 75 μM) were employed compared to those that were suitable for the growth factors in order to show their neuroprotective effects clearly (means ± SEM, n=6). $^{**}p < 0.01$ and $^{***}p < 0.001$ vs. glutamate alone by two-tailed Dunnett's test.
