# Supplementary material for: Systemic treatment with a novel basic fibroblast growth factor mimic small-molecule compound boosts functional recovery after spinal cord injury
Source: PLoS One. 2020 Jul 17;15(7):e0236050. doi: 10.1371/journal.pone.0236050 (PMC7367485; doi:10.1371/journal.pone.0236050)
Supplement: S3 Fig — (PDF) [file pone.0236050.s003.pdf]

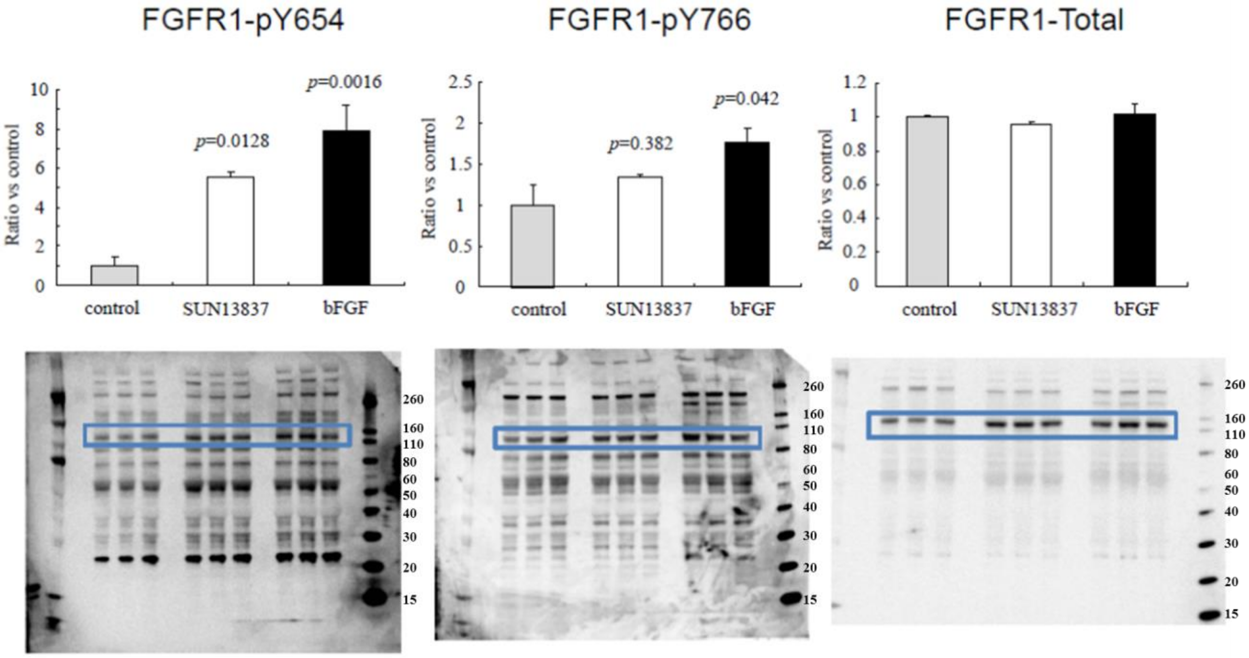


**S3 Fig. Effect of SUN13837 and bFGF on autophosphorylation sites of FGFR1 in rat primary neurons.** The major autophosphorylation sites of FGFR1 have been identified as tyrosine residues 463, 583, 585, 653, 654, 730, and 766 both in vitro and in living cells [41]. We attempted to detect the phosphorylation of Y766 and Y654, which seems to play an important role in activation of FGFR and subsequent cell proliferation. Phosphorylation of intracellular FGFR-1 tyrosine residues in rat primary neuronal cells after treatment with 10 µM SUN13837 and 10 ng/ml bFGF. An expected 110 kDa band was observed for the high molecular mass variant of FGFR1. Western blots and quantification of band intensity is shown for pY654 FGFR-1 (anti-pY654 FGFR1, Abgent) and pY766 (anti-pY766, Abcam) FGFR-1, total FGFR-1 (anti-total FGFR-1, Abcam). Both SUN13838 and bFGF promote Y654 phosphorylation (n = 3, p = 0.013 for SUN13837, p = 0.0016 for bFGF by two-tailed Dunnett's test vs Vehicle). Activation of FGFR-1 proceeds by ligand binding and promotion of autophosphorylation by dimerization.

In that case, the two autophosphorylation sites in the activation loop (Y653/Y654) are essential sites for tyrosine kinase activation [42]. Therefore, it was confirmed that SUN13837 induces activation of FGFR-1 through a process similar to bFGF. Only bFGF enhanced phosphorylation of Y766 (n=3, p = 0.38 for SUN13837, p = 0.042 for bFGF by two-tailed Dunnett's test vs Vehicle) that functions as a binding site for the SH2 domain of phospholipase C-γ (PLC γ) [43, 44] which lead to cell proliferation. Further study of FGFR-1 activation mechanism by SUN13837 is needed, but this point seems to be one of the reason why SUN13837 does not shows cell proliferation activity. Total FGFR-1 expression level did not change both SUN13837 and bFGF.

**References**


41. Furidui CM, Lew ED, Schlessinger J, Anderson KS. Autophosphorylation of FGFR1 kinase is mediated by a sequential and precisely ordered reaction. Molecular Cell. 2006;21: 711-717.

42. Lemmon MA, Schlessinger J. Cell signaling by receptor tyrosine kinases. Cell. 2010;141: 1117-1134.

43. Mohammadi M, Honegger AM, Rotin D, Fischer R, Bellot F, Li W et al. A tyrosine-phosphorylated carboxy-terminal peptide of the fibroblast growth factor receptor (Flg) is a binding site for the SH2 domain of phospholipase C-γ1. Mol Cell Biol. 1991;11: 5068-5078.

44. Bae JH, Lew ED, Yuzawa S, Tome F, Lax I, Schlessinger J. Structural basis underlying a novel mechanism for control of receptor tyrosine kinase signal selectivity. Cell. 2009;138: 514-524.
