# Supplementary material for: Systemic treatment with a novel basic fibroblast growth factor mimic small-molecule compound boosts functional recovery after spinal cord injury
Source: PLoS One. 2020 Jul 17;15(7):e0236050. doi: 10.1371/journal.pone.0236050 (PMC7367485; doi:10.1371/journal.pone.0236050)
Supplement: S4 Fig — (PDF) [file pone.0236050.s004.pdf]

The DiI-impregnated filter was inserted into the sensorimotor cortex of hind limb.

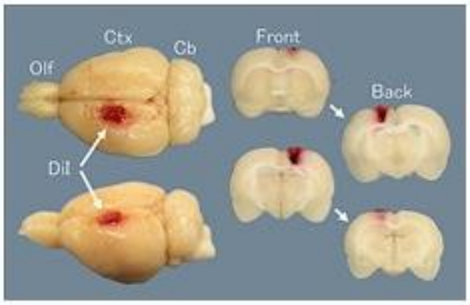

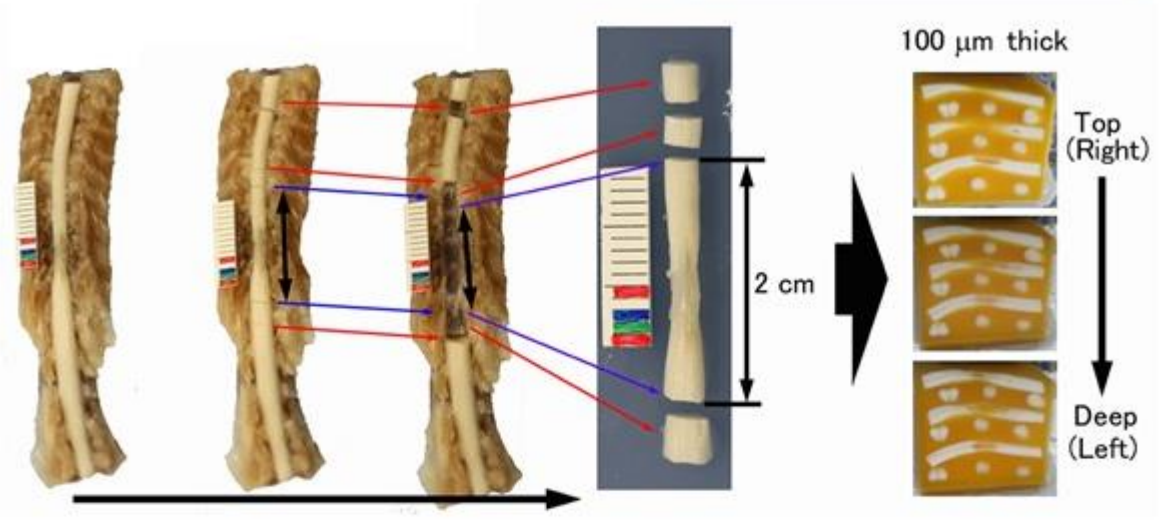

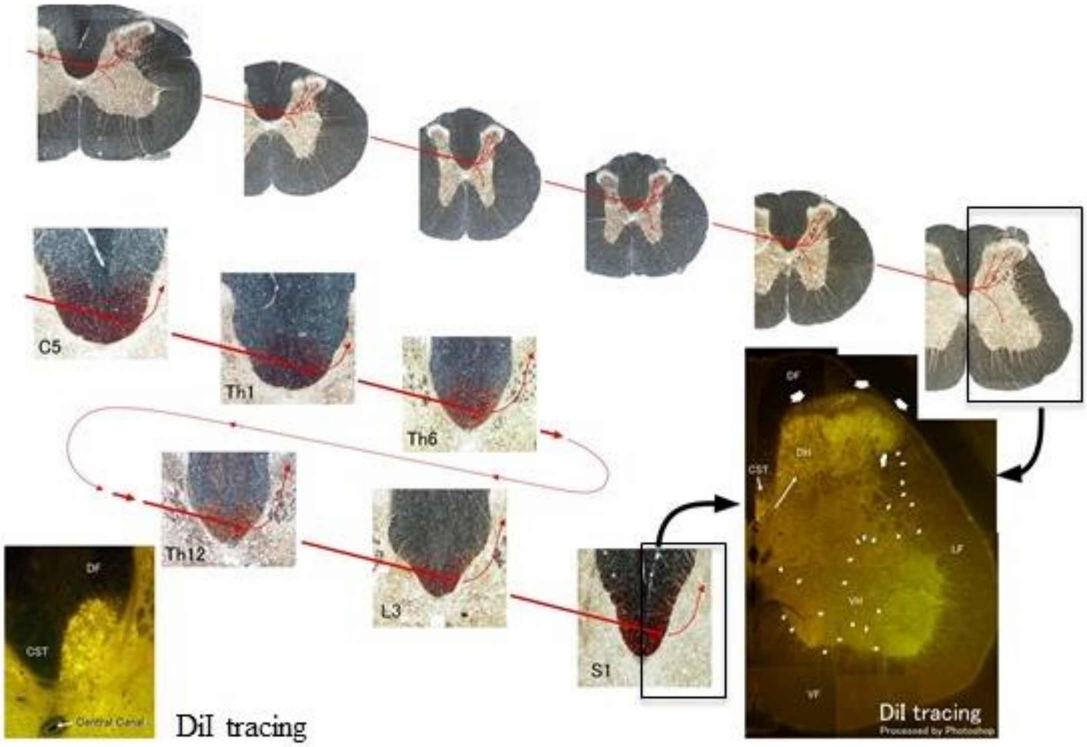

Fig. S4. Schematic drawing of the anterograde axonal tracing method. In order to label cortico-spinal tract axons and axon terminals projecting to the lumbosacral spinal cord, one to four thin strips (less than 0.5 mm in width) of DiI-impregnated PVDF membrane filters were inserted into the hind limb region of the sensorimotor cortex (less than 2 mm in depth), where the cortico-lumbosacral projection neurons were localized. The neurons in the cortical 5th layer were retrogradely labeled following DiI-application to the dorsal lumbosacral spinal cord. Rats were allowed to survive for either one or two weeks. Then, under deep anesthesia, animals were perfused transcardially with saline initially, followed by 4% paraformaldehyde in 0.1 M sodium-phosphate buffer (pH 7.4), and their spines and spinal cords were removed from the rest of the body and post-fixed in the same fresh fixative in a refrigerator (4°C) for several days. The thoraco-lumbo-sacral spinal cord, which contained contusion lesion, were dissected out of the rat spines, divided into 4 segments and then embedded in egg yolk to position each spinal segments in the same manner. That is, spinal cords from three rats were embedded together in a single egg yolk block and the four spinal cord segments from a single animal were positioned in a arranged layout as illustrated (top row: sagittal section of the contusion lesion segment, second row: [left] frontal sections of the post-lesion segment, [middle] the pre-lesion segment, and [right] the remote upper segment; the spinal cords of the other rats were arranged in the same manner). Serial 100 μm thick sections were cut on a microslicer (LinearSlicer Pro7, Dosaka EM Co. Ltd., Kyoto, Japan), collected in phosphate-buffered saline (PBS), and mounted on slide and coversliped with glycerol. Sections were examined carefully and photographed under the epifluorescent microscope (BX51-FL microscope and DP71 digital camera system, Olympus, Japan). In rats untreated with either vehicle or SUN13837, DiI-labeled corticospinal tract axons emerging from the hind limb region of the sensorimotor cortex traveled down along the tract,

decussate and swing dorsally at lower medulla oblongata to enter and descend the deep portion of the contralateral dorsal funiculus, giving off axon terminals toward primarily the dorsal horn. Labeled axons were able to be traced to the caudal end of the spinal cord. Labeled-axons and axon terminals invading into the deep dorsal horn and ventral horn were scanty in number. Anterograde axonal tracing in experimental animals was done in the same way (surgery, survival time, preparation of materials, epifluorescent microscopy, and so forth).
