# Supplementary material for: Systemic treatment with a novel basic fibroblast growth factor mimic small-molecule compound boosts functional recovery after spinal cord injury
Source: PLoS One. 2020 Jul 17;15(7):e0236050. doi: 10.1371/journal.pone.0236050 (PMC7367485; doi:10.1371/journal.pone.0236050)
Supplement: S5 Fig — (PDF) [file pone.0236050.s005.pdf]

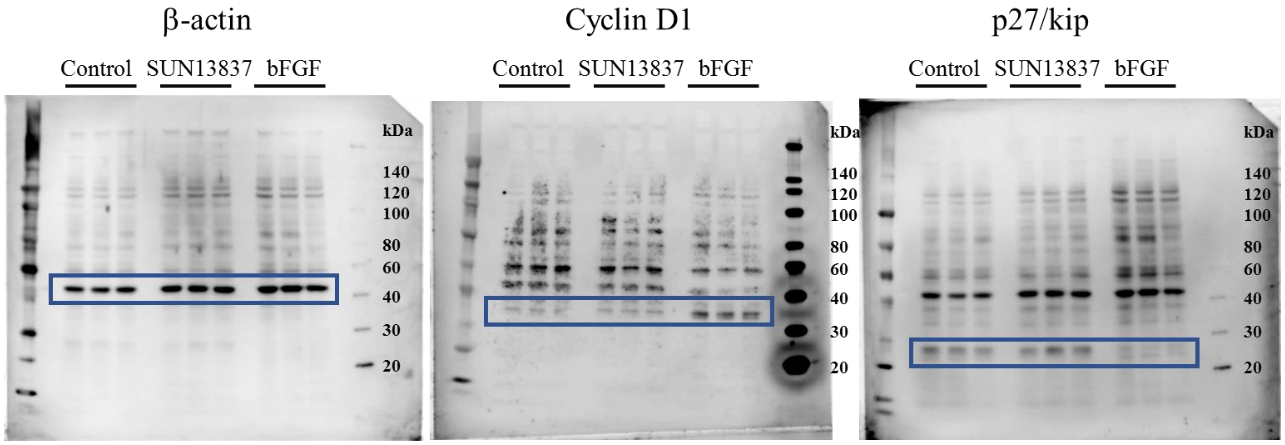

S5 Fig. Immunoblotting membrane images of cyclin D1 and p27 (kip1) protein expression. Swiss 3T3 cells were incubated with 10 μM SUN13837, 10 ng/ml bFGF or HBSS for 16 hrs. Each of three membranes per sample was incubated with anti-cyclin D1, anti-p27/kip 1 and anti-actin primary antibody, respectively. Quantification of immunoblots data are shown in Fig. 3.
