# Supplementary material for: Systemic treatment with a novel basic fibroblast growth factor mimic small-molecule compound boosts functional recovery after spinal cord injury
Source: PLoS One. 2020 Jul 17;15(7):e0236050. doi: 10.1371/journal.pone.0236050 (PMC7367485; doi:10.1371/journal.pone.0236050)
Supplement: S6 Fig — (PDF) [file pone.0236050.s006.pdf]

a.

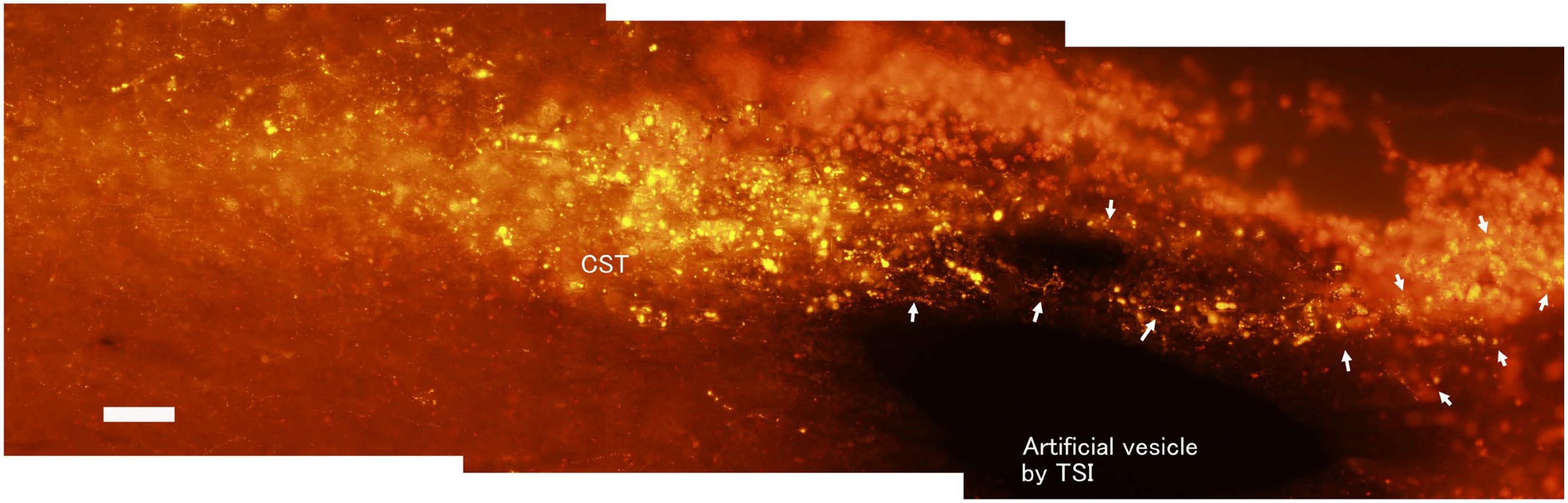

b.

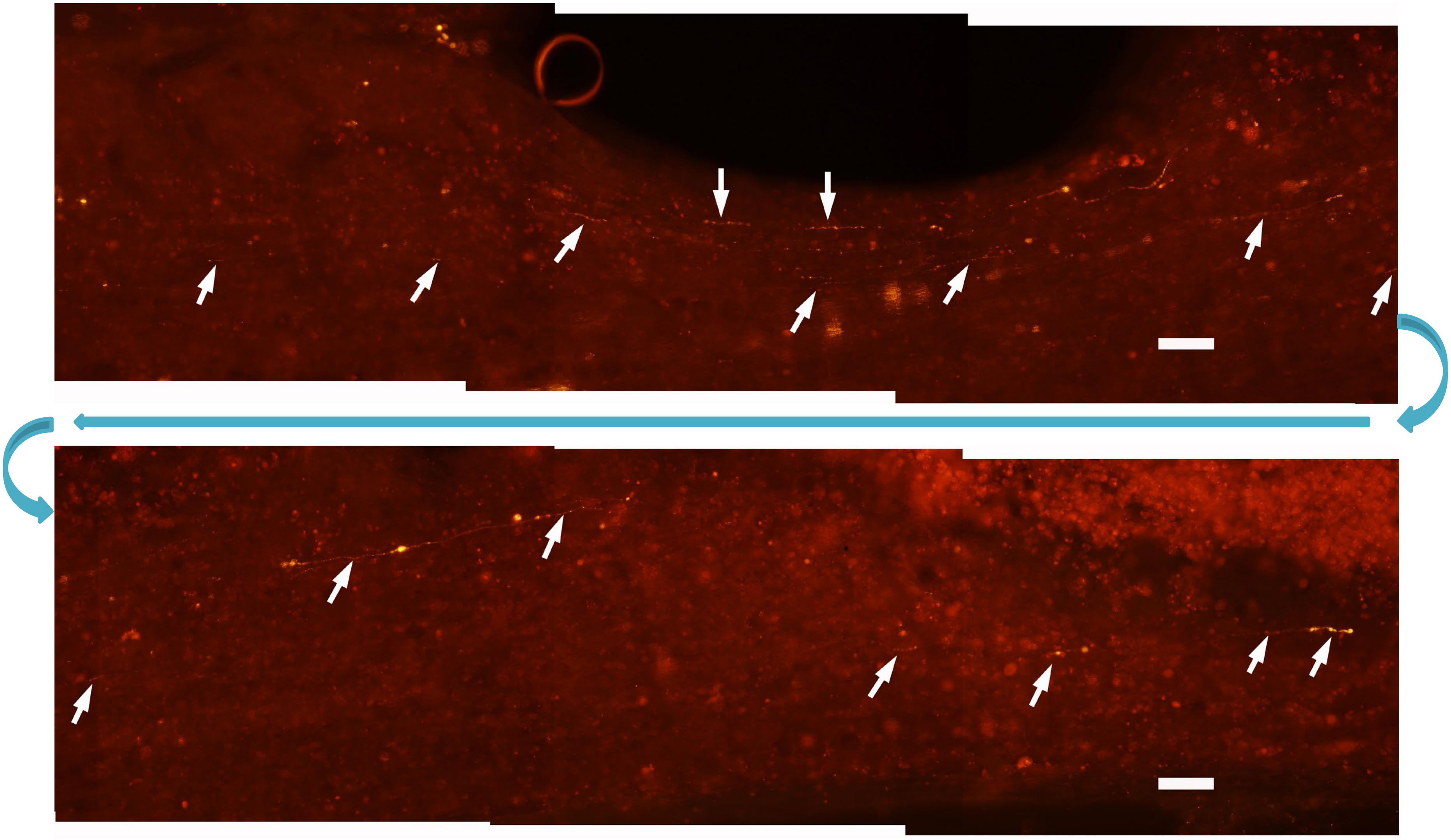

C.

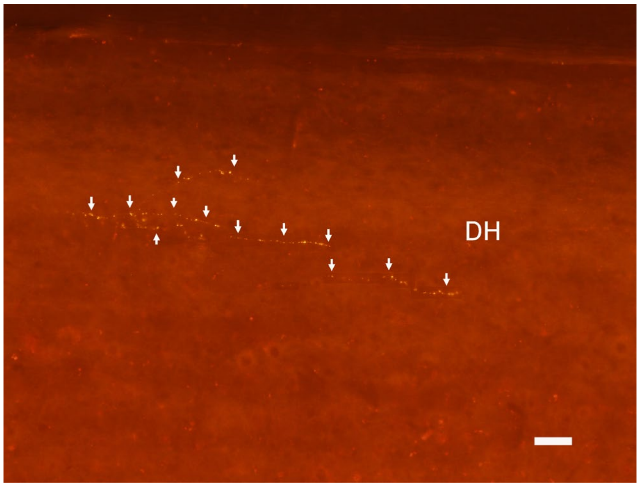

D.

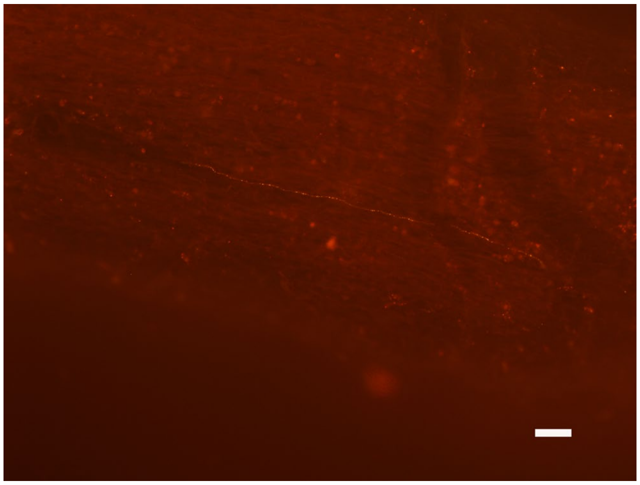

E.

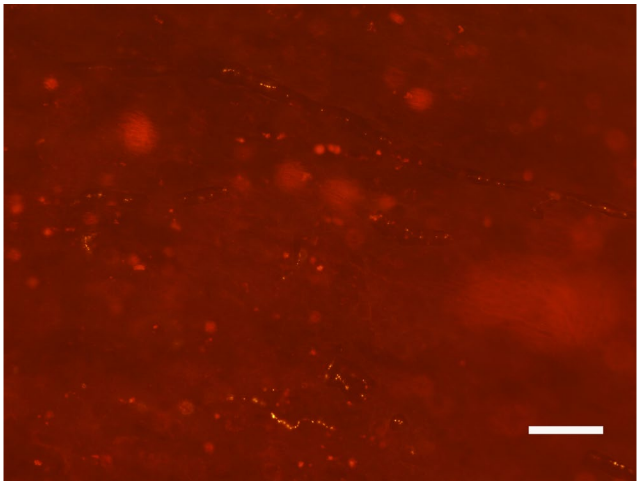

S6 Fig. Magnified axonal tracing image with DiI in rat spinal cord injury model. a. Caudal extention of regenerating axons which pass through the inner bridge surface of the artificial vesicle formed by traumatic spinal injury (TSI). b. DiI-labeled axons seen in the ventral funiculus of the spinal cord in the dorsal horn and lateral funiculus of the lower thoracic spinal cord. We could not confirm such an axonal extention for a long distance on the ventral side in the vehicle-treated group. c. DiI-labeled axons identified in the uninjured dorsal horn (DH) of an intact lumbosacral spinal cord. d. DiI-labeled axons extention to the uninjured spinal cord of post-TSI in the dorsolateral region (caudal side) of the cavity of the TSI part (the type of tissue cannot be specified because it is a cavity damaged tissue) e. DiI-labeled axon that runs and branches in the dorsal horn of uninjured spinal cord of post-TSI.

CST: corticospinal tract, White scale bar represents 50 µm
