# Supplementary material for: Systemic treatment with a novel basic fibroblast growth factor mimic small-molecule compound boosts functional recovery after spinal cord injury
Source: PLoS One. 2020 Jul 17;15(7):e0236050. doi: 10.1371/journal.pone.0236050 (PMC7367485; doi:10.1371/journal.pone.0236050)
Supplement: S7 Fig — (PDF) [file pone.0236050.s007.pdf]

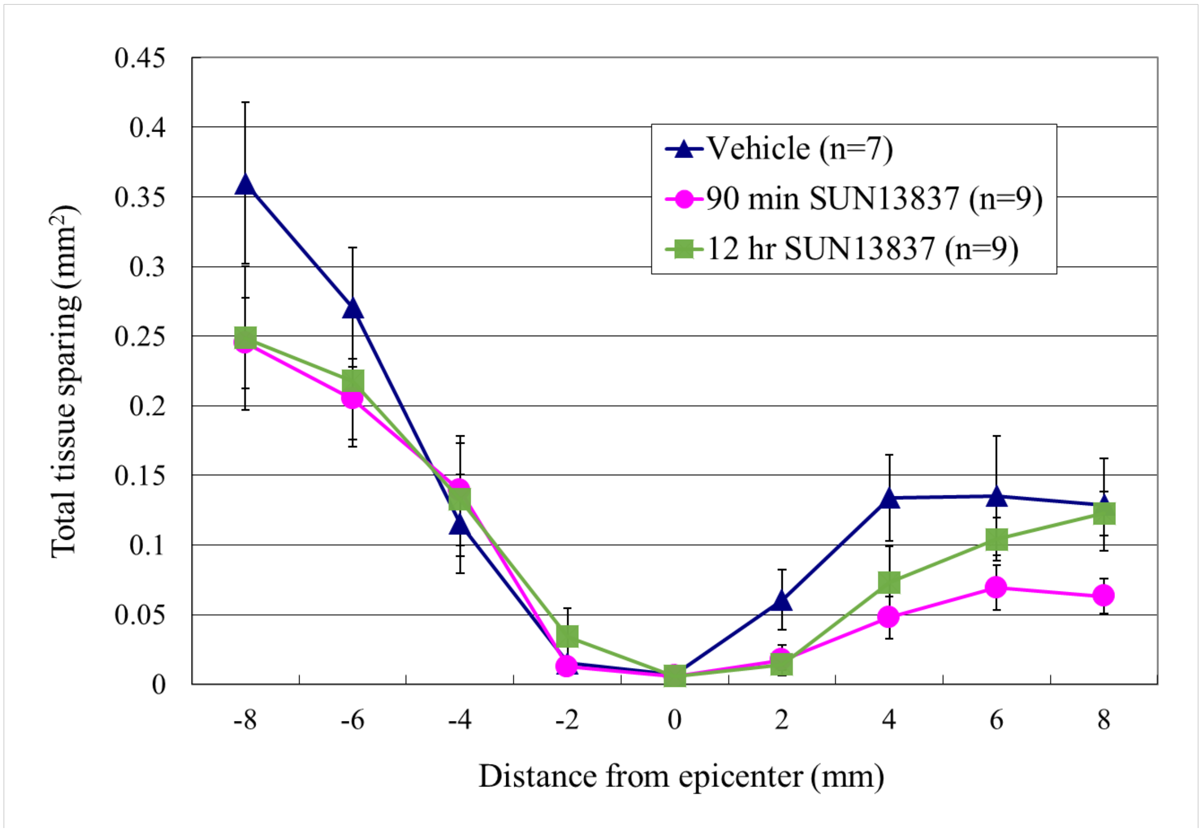

S8 Fig. Effects of SUN13837 treatment on spinal cord tissue sparing. The rat spinal cord injury model was prepared by the method described in the Materials and Methods. SUN13837 (1 mg/kg) or vehicle was administered intravenously via the tail vein at 90 minutes or 12 hours after contusion in a volume of 1 ml/kg once daily for 10 days. Two weeks after SCI, the animals were deeply anesthetized and perfused transcardially with 0.9% saline followed by 4% paraformaldehyde in 0.1 M phosphate buffer. The spinal cords were carefully removed, embedded in paraffin and coronal HE sections were prepared. The spared tissue was measured by ImageJ. Each point is mean ± SEM.
